# Phase Angle and Handgrip Strength Are Sensitive Early Markers of Energy Intake in Hypophagic, Non-Surgical Patients at Nutritional Risk, with Contraindications to Enteral Nutrition

**DOI:** 10.3390/nu7031828

**Published:** 2015-03-11

**Authors:** Riccardo Caccialanza, Emanuele Cereda, Catherine Klersy, Chiara Bonardi, Silvia Cappello, Lara Quarleri, Annalisa Turri, Elisabetta Montagna, Isabella Iacona, Francesco Valentino, Paolo Pedrazzoli

**Affiliations:** 1 Nutrition and Dietetics Service, Fondazione IRCCS Policlinico San Matteo, Viale Golgi 19, 27100 Pavia, Italy; E-Mails: e.cereda@smatteo.pv.it (E.C.); c.bonardi@smatteo.pv.it (C.B.); cappellosilvia@hotmail.it (S.C.); laraquarleri@libero.it (L.Q.); terrianna@libero.it (A.T.); e.montagna@smatteo.pv.it (E.M.); 2 Biometry and Clinical Epidemiology Service, Fondazione IRCCS Policlinico San Matteo, Viale Golgi 19, 27100 Pavia, Italy; E-Mail: klersy@smatteo.pv.it; 3 Pharmacy Unit, Fondazione IRCCS Policlinico San Matteo, Viale Golgi 19, 27100 Pavia, Italy; E-Mail: i.iacona@smatteo.pv.it; 4 Division of Medical Oncology, Department of Hemato-Oncology, Fondazione IRCCS Policlinico San Matteo, Viale Golgi 19, 27100 Pavia, Italy; E-Mails: f.valentino@smatteo.pv.it (F.V.); p.pedrazzoli@smatteo.pv.it (P.P.)

**Keywords:** bioelectrical impedance vectorial analysis, phase angle, handgrip strength, energy intake, hypophagia, nutritional risk, supplemental parenteral nutrition

## Abstract

The assessment of nutritional intakes during hospitalization is crucial, as it is known that nutritional status tends to worsen during the hospital stay, and this can lead to the negative consequences of malnutrition. International guidelines recommend the use of parenteral nutrition (PN) in hypophagic, non-surgical patients at nutritional risk, with contraindications to enteral nutrition. However, to date, there are no published data regarding either energy intake or objective measurements associated with it in this patient population. The aim of the present exploratory methodological study was to evaluate whether phase angle (PhA) and handgrip strength normalized for skeletal muscle mass (HG/SMM) are sensitive early markers of energy intake in hypophagic, non-surgical patients at nutritional risk, with contraindications to enteral nutrition. We evaluated 30 eligible patients, who were treated with personalized dietary modifications and supplemental PN for at least one week during hospitalization. In a liner regression model adjusted for age, gender, basal protein intake and the basal value of each variable, a trend toward improvement of PhA and preservation of HG/SMM was observed in patients satisfying the estimated calorie requirements (*N =* 20), while a significant deterioration of these parameters occurred in those who were not able to reach the target (*N =* 10). The mean adjusted difference and 95% CI were +1.4° (0.5–2.3) (*p =* 0.005) for PhA and +0.23 (0.20–0.43) (*p =* 0.033) for HG/SMM. A significant correlation between PhA and HG/SMM variations was also observed (*r* = 0.56 (95% CI, 0.23–0.77); *p =* 0.0023). PhA and HG/SMM were able to distinguish between hypophagic, non-surgical patients at nutritional risk who satisfied their estimated caloric requirements and those who did not after a one-week personalized nutritional support. Clinical studies are warranted, in order to verify these preliminary observations and to validate the role of PhA variations as early markers of anabolic/catabolic fluctuations.

## 1. Introduction

Nutritional status deterioration is a common feature in hospitalized patients, particularly in those with oncologic diseases, which negatively affects survival, length of stay, morbidity and quality of life [1,2,3,4]. It is known that nutritional status tends to worsen during hospitalization [2,4,5] and that inadequate provision of nutrition support may negatively affect both nutritional status and prognosis [1,6,7].

International guidelines recommend the use of parenteral nutrition (PN) during non-surgical therapy (Grade C), if patients are hypophagic, at nutritional risk, affected by severe iatrogenic gastrointestinal complications or nil by mouth is prescribed for more than five days and enteral nutrition is not feasible [7]. However, to date, there are no published data regarding either energy intake or objective measurements associated with it in hypophagic, non-surgical patients at nutritional risk, with contraindications to enteral nutrition.

Numerous recent studies have proven the prognostic impact of phase angle (PhA) in different clinical settings, and bioelectrical impedance vectorial analysis (BIVA) has been shown to allow the suitable assessment of patients in whom the calculation of body composition fails due to altered hydration [8,9]. In particular, PhA has been shown to be correlated with functional status [10] and predictive of prognosis (mortality, disease progression, incidence of postoperative complications, length of hospital stay) in pancreatic [11], colorectal [12], breast [13], lung cancer [14,15] and cirrhotic patients [16]. Hence, PhA can be considered a reliable prognostic marker and should be used for nutritional assessment and monitoring.

We recently started to use BIVA for routinary nutritional assessment and monitoring of patients who receive nutritional support. In the present exploratory methodological study, we reported the data of 30 hypophagic, non-surgical patients at nutritional risk, with contraindications to enteral nutrition, who were treated with personalized dietary modifications and supplemental PN for at least one week during hospitalization. In particular, our aim was to evaluate whether PhA and handgrip strength normalized for skeletal muscle mass (HG/SMM) are sensitive early markers of energy intake in this patient population.

## 2. Methods

### 2.1. Ethics

The study was approved by the Institutional Ethics Committee of the Fondazione Istituto di Ricovero e Cura a Carattere Scientifico (IRCCS) Policlinico San Matteo (Pavia, Italy) and was conducted according to the Declaration of Helsinki. Written informed consent was obtained for each patient.

### 2.2. Subjects

The subjects were hypophagic, non-surgical patients at nutritional risk, with contraindications to enteral nutrition, consecutively evaluated during the nutritional consultations carried out by the staff of the Nutrition and Dietetics Service in the Clinical Units of the Fondazione IRCCS Policlinico San Matteo (Pavia, Italy).

Inclusion criteria: Nutritional Risk Screening 2002 [17] score ≥3; hypophagia (estimated oral intake <60% of estimated caloric requirements); supplemental PN practicable for at least 6 consecutive days; enteral nutrition contraindicated or not feasible (refusal, nausea, vomiting, diarrhea) according to the physicians’ judgment, based on the severity of symptoms; written informed consent.

Exclusion criteria: age < 18 years; Eastern Cooperative Oncology Group performance status >2 [18]; preadmission home artificial nutrition.

### 2.3. Assessments

The following data were collected: age, sex, diagnosis; body weight (at 1st visit and Day 7; Wunder mechanical weighing scales), 6-month and 1-month previous unintentional body weight loss (at 1st visit), height (at 1st visit), body mass index (BMI) (at 1st visit and Day 7); caloric requirements (estimated multiplying the resting energy expenditure calculated by the Harris–Benedict equation by a correction factor of 1.2–1.5 according to nutritional status and clinical conditions) (at 1st visit); protein requirements (set to 1.3 g kg^−1^ and 1.0 g kg^−1^ body weight for those with BMI <27 kg m^−2^ and BMI ≥27 kg m^−2^, respectively); calorie and protein oral intakes (estimated by 24 h dietary recall) (at 1st visit, Day 3 and Day 7); total energy and protein intakes throughout the treatment period (calculated by the mean values of the 24 h dietary recalls and PN prescriptions); symptoms affecting oral intake (anorexia, dysphagia, odynophagia, dysgeusia, nausea, vomiting and diarrhea) (at 1st visit and Day 7); PhA (evaluated by a BIVA analyzer; NutriLAB, Akern/RJL) (at 1st visit and Day 7); whole-body skeletal muscle mass (SMM) estimated according to Janssen *et al*. [19]; handgrip strength (HG; measured by a digital hand dynamometer; DynEx™, Akern/MD Systems) and handgrip strength normalized for SMM (HG/SMM) [20] (at 1st visit and Day 7); blood tests (glycemia, Na, K, Cl, Ca, Mg, P, triglycerides, total cholesterol, HDL cholesterol, serum creatinine, urea, gamma-glutamyl transferase, alanine transaminase, aspartate transaminase, total bilirubin) (at 1st visit and Day 7); clinical complications (infections, cardiac, renal and respiratory dysfunctions, hypertension, fluid retention, peripheral phlebitis) occurring during the treatment period.

### 2.4. Treatment

Treatment consisted of one-week supplemental PN by multi-chamber bags containing glucose, amino acids, lipids and electrolytes, supplemented with multivitamin and multimineral-trace elements, when indicated. PN was infused either via central or peripheral infusion lines (as available) during 20–24 h per day. It was prescribed in accordance with clinical conditions (hydration status) and biochemical data (baseline electrolyte, glycemic and lipid profiles), in order to satisfy the estimated energy requirements according to the estimated calorie intake at admission. Patients were allowed to continue eating according to individual tolerance; personalized dietary modifications were made according to patients’ preferences and clinical conditions.

### 2.5. Aims

The aims of the study were as follows:

 To assess the change in PhA and HG/SMM in hypophagic, non-surgical patients at nutritional risk, with contraindications to enteral nutrition after a one-week personalized nutritional support.
 To compare the change in PhA and HG/SMM between patients satisfying *vs.* those not satisfying their energy estimated requirements after the one-week personalized nutritional support.
 To assess whether PhA is a marker of HG/SMM in this patient population.

### 2.6. Statistics

Sample size calculation was based on the desired precision of change in PhA. Assuming a PhA standard deviation of change at Day 7 of 1.5° (a value suggested as relevant by our internal preliminary data), with 30 subjects, the 95% confidence interval (95% CI) will extend 0.6° from the expected mean change.

Continuous variables were presented as means and standard deviations (SDs) or medians and interquartile ranges (IQRs). Categorical variables, including also clinical complications (infections, cardiac, renal and respiratory dysfunctions, hypertension, fluid retention, peripheral phlebitis), were described as counts and percentages. Pre-nutritional support characteristics were compared between patients satisfying *vs.* those not satisfying energy estimated requirements by using either the unpaired Student’s *t*-test or the Mann–Whitney U-test (as appropriate) and the Fisher exact test for continuous and categorical variables, respectively. Changes over time in PhA, anthropometry and HG/SMM were assessed by means of general linear models for repeated measures, with the calculation of robust Huber-White standard errors to account for intra-patient correlation, while adjusting for age, gender and basal protein intake. These changes were compared between patients satisfying *vs.* those not satisfying energy estimated requirements with the same model as above, while adjusting for the baseline value of each variable, as well. The correlation of changes in PhA and HG/SMM was assessed through Spearman’s statistic and the 95% CI.

All statistical analyses were performed using STATA 13.1 statistical software (Stata Corporation, College Station, TX). A two-sided *p*-level of <0.05 was adopted as significant.

## 3. Results

Of the 30 patients evaluated, 20 had oncologic diseases, six infectious diseases and four gastrointestinal diseases. The clinical features of the study population according to the satisfaction of estimated energy requirements are reported in Table 1. In particular, the baseline characteristics were substantially similar, with the exception of higher protein intakes and a marginally significant younger age in patients satisfying the estimated protein-calorie requirements.

Energy intake increased in both study groups (*p* < 0.001). A significant deterioration of PhA and HG/SMM was observed in patients who were not able to satisfy the estimated calorie requirements (Table 2).

**Figure 1:**
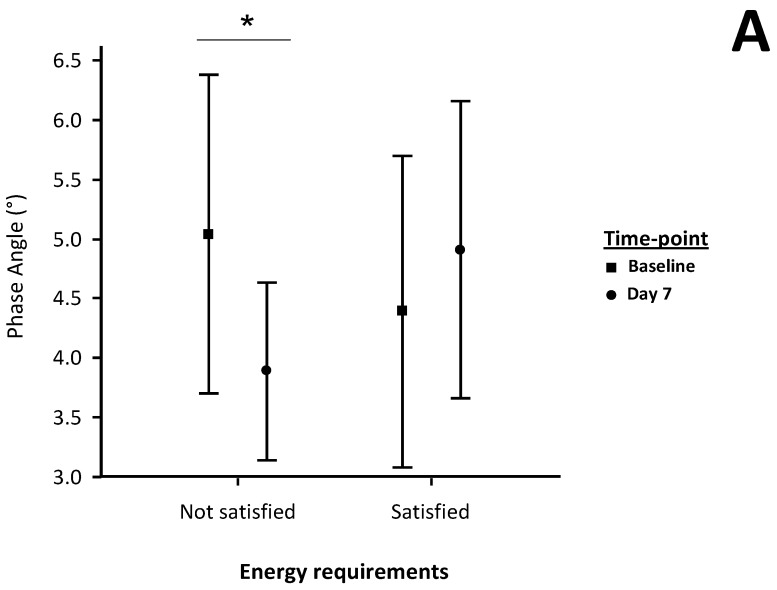

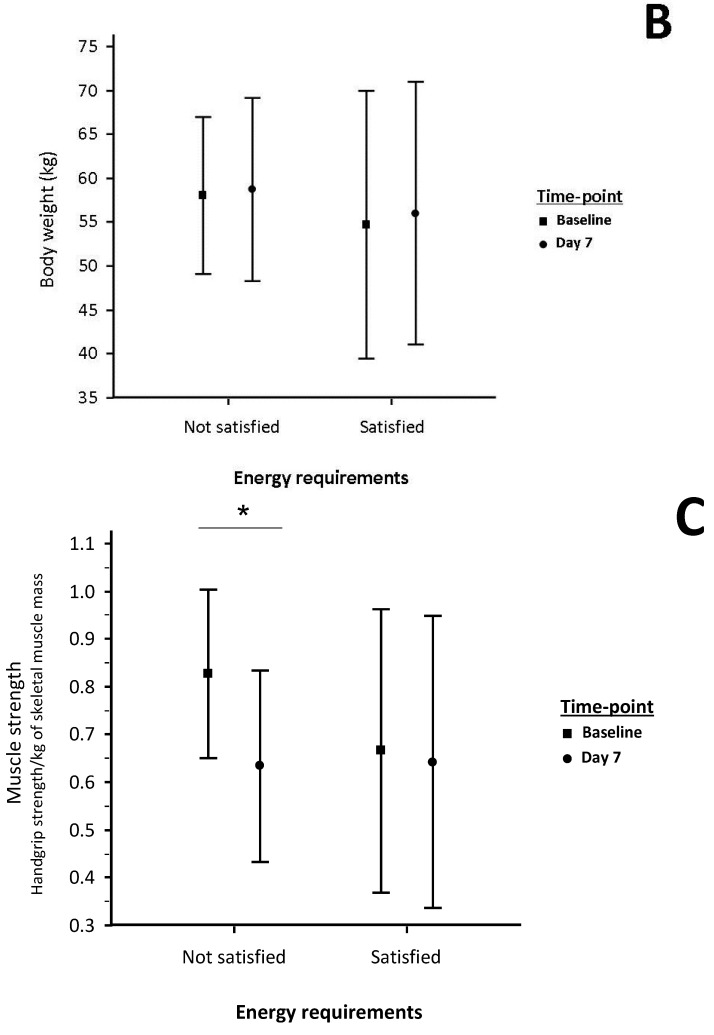
Variations (mean and SDs) in phase angle (**A**), body weight (**B**) and handgrip strength adjusted for skeletal muscle mass (**C**) by satisfaction of estimated energy requirements. * *p*-value ≤ 0.01 (baseline *vs.* end of study).

The mean adjusted differences and 95% CI were +1.4° (0.5 – 2.3) (*p =* 0.005) for PhA and +0.23 (0.20–0.43) (*p =* 0.033) for HG/SMM, respectively, while body weight did not change (Figure 1).

A significant correlation between PhA and HG/SMM variations was also observed (*r* = 0.56 (95% CI, 0.23–0.77); *p =* 0.0023).

**Figure 2:**
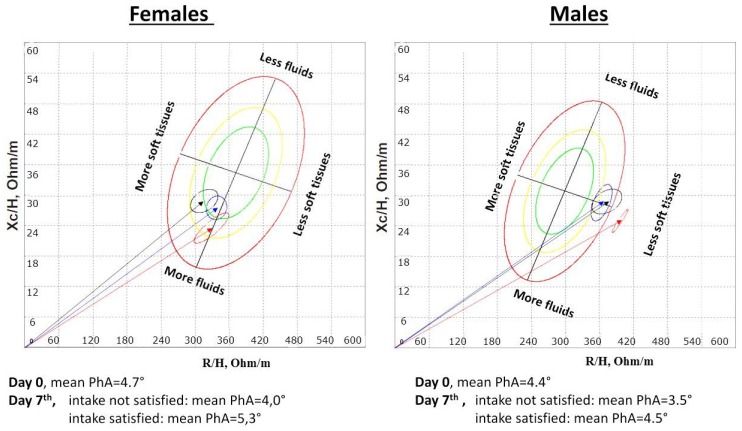
Mean impedance vectors and confidence ellipses on the bioelectrical impedance vectorial analysis (BIVA) nomogram by sex and satisfaction of estimated energy requirements (blue color = overall population at baseline assessment; red color = patients not satisfying estimated energy requirements at Day 7; black color = patients satisfying estimated energy requirements at Day 7).

The changes in mean impedance vectors and confidence ellipses on the BIVA nomogram by sex and satisfaction of estimated energy requirements are reported in Figure 2.

At Day 7, we registered one episode of hyperglycemia (blood glucose > 180 mg dL^−1^), three episodes of hypertriglyceridemia (serum triglycerides > 300 mg dL^−1^), one episode of fluid retention, seven episodes of hyponatremia (*n =* 6, Na <135 mEq L^−1^ and >130 mEq L^−1^; *n =* 1, Na < 130 mEq L^−1^), one episode of hypokalemia (K = 3.2 mEq L^−1^) and one episode of hypomagnesaemia (Mg = 1.2 mg dL^−1^). All of the other blood tests improved or did not change.

**Table 1:** Clinical features of the study population according to the satisfaction of estimated energy requirements.

Variable	Overall	Satisfied	Not Satisfied	*p*-Value
	(*N* = 30)	(*N* = 20)	(*N* = 10)	
***Baseline***				
**Gender** (male), *N* (%)	15 (50)	9 (45)	6 (60)	0.700
**Age** (years), mean (SD)	63.0 (13.7)	59.8 (13.3)	69.4 (11.2)	0.058
**Baseline body weight** (kg), mean (SD)	55.8 (13.4)	54.7 (15.2)	58.0 (8.9)	0.363
**Baseline body mass index** (kg·m^−2^), mean (SD)	20.2 (3.9)	20.0 (4.3)	20.6 (2.5)	0.619
**6-month weight loss** (%), mean (SD)	11.8 (6.4)	12.1 (5.9)	11.1 (7.1)	0.728
**Phase Angle**, mean (SD)	4.6 (1.3)	4.4 (1.3)	5.0 (1.3)	0.223
**Body cell mass**, mean (SD)	19.7 (6.9)	19.3 (7.6)	20.6 (4.9)	0.602
**Whole-body skeletal muscle mass** (kg), mean (SD)	22.1 (4.9)	22.3 (5.0)	21.5 (4.5)	0.680
**Handgrip strength** (kg), mean (SD)	16.2 (7.7)	15.2 (8.0)	18.5 (5.9)	0.277
**Adjusted-handgrip strength ^a^**, mean (SD)	0.69 (0.31)	0.67 (0.29)	0.83 (0.17)	0.096
**Administration of PN** (central infusion line), N (%)	13 (43)	11 (55)	2 (20)	0.119
**Estimated energy requirements** (kcal·day^−1^), mean (SD)	1820 (270)	1805 (291)	1846 (204)	0.667
(kcal (kg·day^−1^)), mean (SD)	33.4 (4.5)	34.2 (5.0)	32.1 (2.3)	0.150
**Estimated protein requirements** (g (kg·day^−1^)), mean (SD)	1.3 (0.1)	1.3 (0.1)	1.3 (0.1)	1.00
**Baseline energy intake** (kcal·day^−1^), mean (SD)	608 (487)	690 (493)	446 (405)	0.179
(kcal (kg·day^−1^)), mean (SD)	11.7 (10.8)	13.7 (11.2)	7.8 (8.1)	0.129
**Percentage of requirements** (%), mean (SD)	33 (27)	38 (27)	24 (23)	0.185
**Baseline protein intake** (g·day^−1^), mean (SD)	24.9 (22.8)	30.3 (23.5)	14.3 (15.1)	0.040
(g (kg·day^−1^)), mean (SD)	0.49 (0.53)	0.61 (0.57)	0.25 (0.30)	0.040
**Percentage of requirements** (%), mean (SD)	38 (40)	47 (43)	19 (23)	0.037
***After the one-week personalized nutritional support***				
**Final energy intake** (kcal(kg day^−1^)), mean (SD)				
Total	36.9 (12.5)	42.6 (11.2)	25.5 (2.1)	<0.001
Percentage of requirements	111 (30)	127 (23)	80 (6)	<0.001
Oral	16.0 (12.0)	22.2 (9.2)	3.5 (3.2)	<0.001
Supplemental PN	20.9 (6.9)	20.4 (7.8)	22.0 (3.8)	0.462
**Final protein intake** (g/(kg day^−1^))				
Total	1.46 (0.57)	1.71 (0.54)	0.98 (0.14)	<0.001
Percentage of requirements	117 (48)	138 (45)	76 (11)	<0.001
Oral	0.70 (0.56)	0.98 (0.45)	0.15 (0.14)	<0.001
Supplemental PN	0.76 (0.27)	0.73 (0.31)	0.83 (0.14)	0.253

^a^ Handgrip strength normalized for whole-body skeletal muscle mass estimated according to Janssen *et al.* [18]; PN, parenteral nutrition.

**Table 2:** Clinical parameters of the study population after the one-week personalized nutritional support.

Endpoint	Overall (*N =* 30)			Patients Not Satisfying Requirements (*N =* 10)			Patients Satisfying Requirements (*N =* 20)		
	Mean (SD) ^a^	Δ (95% CI) ^b^	*p*-Value ^c^	Mean (SD) ^a^	Δ (95% CI) ^b^	*p*-Value ^c^	Mean (SD) ^a^	Δ (95% CI) ^b^	*p*-Value ^c^
***Phase angle*** (°)	4.6 (1.2)	−0.02 (−0.3, 0.2)	0.875	3.9 (0.8)	−0.7 (−1.1, −0.3)	0.004	4.9 (1.3)	0.3 (−0.04, 0.7)	0.076
***Body weight*** (kg)	56.9 (13.5)	0.4 (−0.8, 1.5)	0.507	58.7 (10.5)	0.8 (−0.8, 2.4)	0.277	56.0 (15.0)	0.2 (−2.8, 3.3)	0.872
***HG/SMM***	0.64 (0.28)	−0.04 (−0.08, 0.001)	0.057	0.63 (0.20)	−0.11 (−0.18, −0.04)	0.010	0.64 (0.31)	−0.01 (−0.07, 0.06)	0.778

Abbreviations: SD, standard deviation; HG/SMM, handgrip strength normalized for whole-body skeletal muscle mass estimated according to Janssen *et al.* [18]. ^a^ Value at the end of study; ^b^ adjusted (age, gender, basal protein intake and baseline value of the study variable) change at the end of study *vs.* baseline; ^c^ *p*-value for comparison (paired data) between baseline and end of study.

## 4. Discussion

This exploratory methodological study provides evidence that PhA and HG/SMM are sensitive early markers of energy intake and that their variations are correlated in hypophagic, non-surgical patients at nutritional risk, with contraindications to enteral nutrition, treated with supplemental PN.

Preliminary experiences in small samples of malnourished pancreatic cancer patients showed that BIVA parameters may improve with supplemental PN [21,22]. In particular, at the group level, an improvement was observed in PhA (+10%), body cell mass (+10%), extracellular mass and cell content. At the individual level, improvement or stabilization in the aforementioned parameters was observed in about 50% and 35% of patients, respectively. However, in these studies, treatment initiation and/or duration were heterogeneous. In our overall population, PhA improved or was stabilized in 60% of patients, while this occurred in up to 80% of those satisfying their energy requirements. Furthermore, the maintenance or improvement of functional status as assessed by HG/SMM was registered in 67% of all patients and 75% of those satisfying their energy requirements.

Nevertheless, being an exploratory methodological study, the clinical relevance of our data is limited. The main limitation is, according to the nature of the study design, the lack of a control group, which did not allow ascertaining whether the patients who satisfied their requirements were healthier than those who were not able to reach the caloric target.

Although exploratory, our findings are of interest, as they show that PhA and HG/SMM were able to distinguish between patients who satisfied their estimated caloric requirements and those who did not reach the caloric target after the one-week personalized nutritional support. The adequacy of energy provision during hospitalization is crucial to prevent the negative consequences of malnutrition [1], particularly with regards to a population of fragile patients at nutritional risk, in whom the nutritional support strategies are limited. Hence, the early detection of calorie deficiency by objective, quick, non-invasive and low-cost measurements, such as PhA and HG/SMM, may represent a significant advantage, as it should overcome the limitations related to the usual methods of dietary intake estimation, such as 24 h recall and food diaries, which rely on patients’ compliance and trustworthiness [23], thus allowing the optimization of nutritional supply. Besides, the significant correlation between PhA and strength variations according to energy intake is interesting, as it may indicate that effective nutritional interventions are likely to result in better functional status.

Another noteworthy observation of our study is that the difference in total calorie-protein intake between patients satisfying or not the estimated requirements was due to oral intake. Taking into consideration the potential clinical relevance of the outcomes, it could be hypothesized that, in future clinical trials, supplemental PN prescription should be reassessed even during the first week of treatment, according to early dietary intake variations, in order to satisfy the estimated requirements in patients who are not able to effectively increase oral intake, as a consequence of their clinical conditions.

According to the changes in mean impedance vectors and confidence ellipses on the BIVA nomogram, the adequacy of energy intake was reflected by the containment of vector pattern derangements in males and vector pattern improvement in females who satisfied the estimated caloric requirements. These differences could be due to males’ higher muscle mass content and faster metabolic changes, but it remains unclear at the moment if multivariate analyses showed no independent effect of gender on changes in both PhA and HG/SMM.

No significant differences in weight were reported, both between baseline and Day 7, in the whole sample and between groups by satisfaction of requirements. This underlines that weight variations cannot be considered a reliable early marker of energy intake, but should be taken into consideration only to identify fluid overload during artificial nutrition. On the other hand, PhA variations were shown to be able to discriminate between patients satisfying or not the caloric requirements and to be significantly correlated with HG/SMM variations. Compared to the assessment of HG/SMM, PhA measurement may be preferable for monitoring energy intake, as it is less influenced by patients’ compliance and characterized by better accuracy and repeatability [8].

Serum prealbumin is a recognized biochemical marker usually used for short-term nutritional monitoring, which was shown to be associated not only with calorie intake, but also with inflammation, kidney and liver function [24]. Compared to serum prealbumin, PhA presents the advantage of being independent of the inflammatory state [25], so it could be considered a more reliable marker compared to the usual nutritional parameters for the short-term monitoring of energy intake.

Finally, another issue that deserves further consideration and possibly standardization in future clinical trials is that of the contraindications to enteral nutrition. Besides refusal of treatment, we considered enteral nutrition contraindicated or not feasible according to our clinical judgment, based on the severity of symptoms, which could be worsened by enteral support. However, it should be acknowledged that in the absence of standardized criteria, contraindications to enteral nutrition are based on the physicians’ judgment, which, in the end, is arbitrary and may result in being controversial.

## 5. Conclusions

This methodological exploratory study showed that PhA and HG/SMM are sensitive early markers of energy intake and that their variations are significantly correlated in hypophagic, non-surgical patients at nutritional risk, with contraindications to enteral nutrition.

Clinical studies are warranted in order to verify these preliminary observations and to validate the role of bioimpedance PhA variations as early markers of anabolic/catabolic fluctuations.
